# AlphaFold can help African researchers to do cutting-edge structural biology

**DOI:** 10.1038/d41586-026-00072-3

**Published:** 2026-01-01

**Authors:** Emmanuel Nji

**Affiliations:** BioStruct-Africa, Nairobi, Kenya

**Keywords:** Structural biology Machine learning, Drug discovery

## Abstract

Structural biology is essential for understanding diseases and for developing drugs and vaccines. Africa has few specialists in this field, owing to limited infrastructure, training and mentorship opportunities — despite the efforts of non-profit organizations such as BioStruct-Africa, which I co-founded.

The advent of artificial-intelligence tools such as AlphaFold that can predict protein structures offers a path to bridging disparities, by reducing the need for the expensive experimental equipment. To take advantage, organizations such as ours need sustained investment in training, infrastructure access and open-science platforms.

In 2024, BioStruct-Africa ran a pilot workshop to train 20 researchers in using AlphaFold. In 2025, we trained 100 participants. These scientists can now investigate protein structures linked to malaria, HIV/AIDS, tuberculosis and neglected African diseases.

Our goal is to train and mentor 100 scientists annually for the next decade. This will produce a community of 1,000 Africa-based structural biologists who can work on diseases of major concern to the continent, and who can act as mentors for future generations. Support from funders, scientific societies and technology firms is essential to see the project through and empower African researchers to contribute fully to scientific discovery.

## Related links

Africa finally has its own drug-regulation agency — and it could transform the continent’s health https://www.nature.com/articles/d41586-025-03637-w

Better data sets won’t solve the problem — we need AI for Africa to be developed in Africa https://www.nature.com/articles/d41586-024-03988-w

